# Fowl adenovirus strains 1/A and 11/D isolated from birds with reovirus infection

**DOI:** 10.1371/journal.pone.0256137

**Published:** 2021-08-19

**Authors:** Jowita Samanta Niczyporuk, Wojciech Kozdrun, Hanna Czekaj, Natalia Stys-Fijol

**Affiliations:** Department of Poultry Diseases, National Veterinary Research Institute, Pulawy, Poland; University of Nicolaus Copernicus in Torun, POLAND

## Abstract

Inclusion body hepatitis (IBH) is, in some cases, a fatal disease affecting fowl by adenovirus strains which are subdivided into 5 species (A-E). In the current study, we investigated sequences from the Loop L1 region of the hexon gene of sequences of adenovirus field stains 1/A and 11/D isolated from a poultry flock co-infected with IBH and avian reoviruses ARVs. In early 2021, an epidemiologic survey highlighted the coinfection adenoviruses with other viruses (orthoreovirus infection) as being particularly deleterious within the poultry industry. Here, we investigated the Loop L1 HVR1-4 region of the hexon gene with relative synonymous codon usage (RSCU) designation and RSCU inclusive of all the mutations. These are the first results that have been presented on fowl adenovirus species A and D with simultaneous reovirus infection in 38-days old broiler chickens in Poland.

## Introduction

In poultry farms, viral coinfections are becoming increasingly frequent. Adenovirus infections are often described as coinfections involving viruses such as infectious bursal disease virus (IBDV) [1], avian reoviruses (ARVs) [2], Marek’s disease virus (MDV) [3, 4], and chicken infectious anaemia virus (CIAV) [1, 5–7]. Fowl adenoviruses (FAdVs) are double-stranded DNA viruses of the adenovirus genus of the *Adenoviridae* family. Further, the different types of adenoviruses (FAdV-1-8a-8b-11) are represented by five species (FAdV-A-E) which frequently exist in poultry flocks [8–11]. Whilst commonly non-pathogenic, under specific conditions, FAdVs can induce disease and other pathological syndromes/disorders [12]. The clinical outcomes of pathogenic coinfections are usually well evaluated; however, a clear explanation of the mechanisms shaping the complex interactions that take place between infectious agents is often lacking.

Inclusion body hepatitis (IBH) is an emerging disease that has detrimental economic significance for the global poultry industry [13–21]. IBH is an acute, viral infection that affects broiler chickens between 2- and 7-weeks of age. The disease appears suddenly and has a short clinical course (4–5 days) and is associated with increased mortality of 1–20% [14, 18, 22, 23] however, mortality may exceed 30–40% [24, 25]. In infected chickens, pale and swollen foci of extensive liver necrosis, as well as the presence of basophilic inclusion bodies in hepatocytes, are characteristic of adenovirus infection [26]. The pathogenesis of IBH is not clearly understood, however, it can be correlated with multiple factors including the host species, adenovirus type/species, specific environmental conditions, or other pathogens such as viruses, bacteria or fungi [14, 24, 27, 28].

Coinfections have been well described in humans, animals, and birds [29, 30]. Viral coinfections, especially with immunosuppressive agents in poultry flocks, have always had a detrimental role in poultry immunity. Several studies have assessed the presence of two or more viral pathogens in poultry and have highlighted the decreasing host immunity status elicited during coinfection [24, 30, 31]. Adenovirus infections may spontaneously cause the disease contribute to a modulation in mechanisms of immunity in chickens, or can be related or may complicate or cause disease with other viruses such as: IBDV, ARVs, CIAV, and MDV. Further, the increasingly accelerated spread of these viruses within the poultry sector has exacerbated fears for the economic consequences of coinfection. Many *in vivo* studies have assessed the clinical severity of the signs as well as the development of the macroscopic/microscopic lesions in infected poultry. Further investigation of viral coinfections will allow for an improved understanding of the biological and economic consequences of coinfections [3, 32–35]. With the evolution and advancement of molecular methods, more and more viruses are being identified and characterized. Several *in vitro* and *in vivo* studies that have assessed coinfections with adenoviruses and Marek’s disease virus have been reported [33]. In poultry, a significant percentage of different FAdVs are pathogenic [1, 36]. Avian adenovirus (6/E, 7/E and 8/E) have been reported to cause IBH in Australia and New Zealand. Saudi Arabia investigated type/species 2/D and 6/E, and newly shifted from 4/C to 8b/E strains indicated in South Korea [37] and other parts of the world [12, 16]. Additionally, researchers from India have reported the presence of avian adenoviruses such as 2/D, 5/B, 6/E, 7/E and 12/D in addition to type/species 4/C and 8/E [38]. In Pakistan, many strains of FAdV of type FAdV-4 have been isolated from broiler flocks with HPS [26].

In this study, we present the results of viral coinfection in a broiler flock. We assessed broiler chickens clinically suspected to have IBH as well as being coinfected with avian reoviruses. Avian reoviruses (ARVs) belong to the *Reoviridae* family replicates in the gut of birds. Pathogenic strains of ARVs indicated in our studies affect tendons causing locomotory disturbances with characteristic clinical manifestation and lesions in liver/spleen of infected birds. Using molecular methods, we aimed to better characterize this coinfection. Further, we discuss the main limitations that make the interpretation of the obtained results difficult.

## Materials and methods

### Clinical signs of infected chickens and sample collection

The analysis were performed on the tissues of sick birds and were fully compliant with the rules of the local ethics committee in Lublin, Poland. The samples were collected in March 2021 from a single broiler flock located in the Kujawsko-Pomorskie voivodeship region in northern Poland (GPS location: latitude 53°05’ North, geographic longitude 18°14’ East). The affected flock was kept in an all-in, all-out management system and contained approximately 10,000 ROSS 308 broiler chickens. Initially, infected chickens were selected based on a depression in weight gain connected with poor feed conversion and hepatitis disorders. Sick chickens adopted a crouching position with ruffled feathers. No respiratory symptoms were noted. Chickens suspected of adenovirus and reovirus infection were collected (10 chickens) and transported to National Veterinary Research Institute for detailed examinations. Necropsy revealed indicative suspicion of IBH infection that the birds had pale and discoloured liver tissue hepatomegally, splenomegally and pale kidneys.

### Chicken embryo fibroblast (CEF) cell culture

CEF cultures were prepared using 11-day-old SPF chicken embryos (Lohmann, Germany) as previously published by Niczyporuk, 2012 [33]. The cells were cultured in Eagle’s medium (MEM) that was supplemented with 10% of bovine serum and 0.1% of an antibiotic mixture (Antibiotic–Antimycotic, Gibco). A monolayer of CEF cells for virus isolation was cultured after about 48 h with the cells incubated at 37.5°C.

### Isolation of adenoviruses

After the homogenization of samples from visceral organs (liver, spleen, gizzard and intestinum), triple freeze, and thaw, centrifugation, and filtering through the Millipore filter of diameter 450 nm, the CEF monolayer was inoculated with the collected materials. The inoculated cultures were incubated at 37.5°C and observed daily via light microscopy. The fifty percent tissue culture infective doses (TCID 50) was calculated using the Reed and Muench model [39].

### DNA extraction

Total DNA was extracted directly from the CEF cultures that were inoculated with homogenates prepared from internal organs. DNA extraction was performed using DNA Mini Kit (Qiagen, Germany) according to the manufacturer’s procedure. Purified DNA samples were stored at -20°C for subsequent analysis.

### Fowl adenovirus reference strain controls

DNA obtained from the reference strain type/species FAdV-1/A (Charles River, US) was used as the positive control. DNA isolated from non-infected CEF cells was used as the negative control in molecular analysis.

### Fowl adenovirus field strains

The G018-21-Liver-11/D strain (GenBank accession number MW741563) was isolated from the liver, G018-21-Gizzard-11/D strain (GenBank accession number MW741564) was isolated from the gizzard, G018-21-Intestinum-11/D strain (GenBank accession number MW741565) was isolated from the intestines, and the G018-21-Spleen-1/A strain (GenBank accession number MW741566) was isolated from the spleen of 38-days old broiler chickens and have been used for the studies.

### Other strains

To detect reovirus strains (ARVs), the RNA of collected samples were extracted using QIAamp® cador® Pathogen Mini Kit (Qiagen, Germany).

### PCR for the identification of the hexon gene of adenovirus strain

PCR for the amplification of FAdVs, specific sequence of the L1 loop region of the hexon gene to produce a PCR product of 830 bp in size, as described previously by Niczyporuk, 2018 [34] have been used.

### RT-PCR for the identification of the σNS gene of reovirus strains

RT-PCR for the reverse transcription and amplification of reovirus strains was based on the sequence of S4 region of ARVs genome coding non-structural σNS protein and was performed as described previously by Woźniakowski, 2014 [40].

### Genome sequence analysis of FAdVs strains

**Fig 1:**
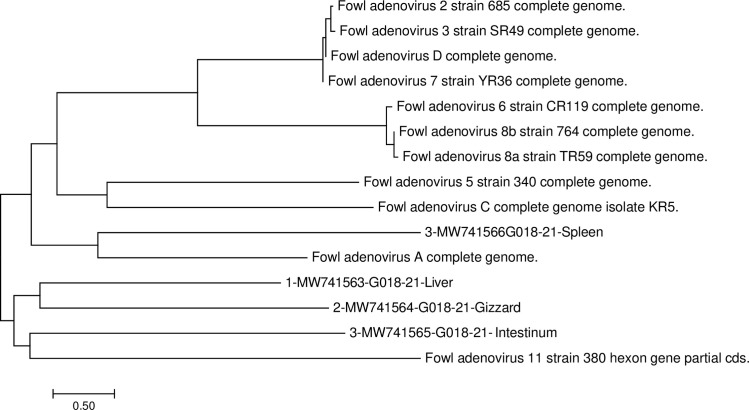
Phylogenetic tree. The phylogenetic tree was based on derived amino acid sequences of the loop L1 region of the hexon gene of adenovirus strains. Isolated strains have been designated as: G018-21-Liver-11/D strain (GenBank accession number MW741563), G018-21-Gizzard-11/D strain (GenBank accession number MW741564), G018-21-Intestinum-11/D strain (GenBank accession number MW741565), and the G018-21-Spleen-1/A strain (GenBank accession number MW741566). The phylogenetic tree was rooted by the 11 reference adenovirus strain sequences obtained from database GenBank (NCBI).

Four sequences of the Loop L1 HVR1-4 region of the hexon gene have been derived and were aligned and compare with reference hexon gene sequences obtained from GenBank, National Center for Biotechnology Information (NCBI) database representing twelve fowl adenovirus type/species A-E with the International Committee on Taxonomy of Viruses, ICTV classification system. All strain sequences which have been used in phylogenetic examinations are presented in Fig 1. The MEGA 7.0.26 version (7170509-x86_64) software was used to perform the alignment. The phylogenetic tree and evolutionary taxa were inferred using the Neighbor-Joining method [41]. The phylogenetic tree with the sum of branch length = 24.35747397 is presented (Fig 1). The phylogenetic tree was drawn to scale with branch lengths in the same units as those of the evolutionary distances used to infer the phylogenetic tree. The evolutionary distances were computed using the Maximum Composite Likelihood method [42] and are in the units of the number of base substitutions per site. Evolutionary analyses were conducted in MEGA7 [43].

### Relative Synonymous Codon usage of FAdVs strain sequence analysis

All field and reference strain sequences were examined under the frequencies with averages over all taxa. The values of RSCU were determined by using MEGA 7.0 [44].

### Analysis of codon composition

Analysis of the codon composition of the examined strain sequences isolated from broiler chickens was indicated and clearly presented [43, 45–47].

### Tajima’s neutrality test

The statistical analysis was performed on 15 examined nucleotide sequences of fowl adenovirus strains. The codon positions at 1^st^ + 2^nd^ + 3^rd^ and noncoding were examined. Positions containing missing data or some invalid sequences have been removed. The abbreviations were as follows: (m) number of sequences, (n) total number of sites, (S) number of segregating sites, (*ps*) Sin, *ps/al (*φ), nucleotide diversity, (π), and the Tajima’s test statistic (D) [48]. Evolutionary analyses were conducted in MEGA7 [43].

## Results

### Virus identification

Four adenovirus strains represented by type/species: one strain 1/A, and three strais represented type/species 11/D were identified in the spleen and gizzard, intestines, and liver of infected chickens respectively. The coinfection of reovirus strains was confirmed in the liver and spleen of affected birds.

### Phylogenetic analysis

Four adenovirus strain sequences from the FAdV Loop L1 region of the hexon gene were obtained from the internal organs of clinically infected broiler chickens from a single flock. The phylogenetic tree constructed using the Neighbor-Joining method (Fig 1) was used as a comparison with the reference strain sequences derived from the GenBank (NCBI) database. These field strains represented one FAdV species, respectively: (MW741563) -G018-21-Liver-D/11, (MW741564) -G018-21-Gizzard-D/11, and (MW741565) -G018-21-Intestinum-D/11 presented a 95% similarity with the reference sequence of FAdV 11/D strain 380. This strain created one visible branch. However, the percentage nucleotide identity based on pairwise comparison of the Polish strain (MW741566) -G018-21-Spleen-A/1 with reference sequence adenovirus strain FAdV A complete genome sequence, showed 98% identity. This strain created a separate branch that was completely phylogenetically distant from other field and reference strain sequences.

### Analysis of codon composition

**Fig 2:**
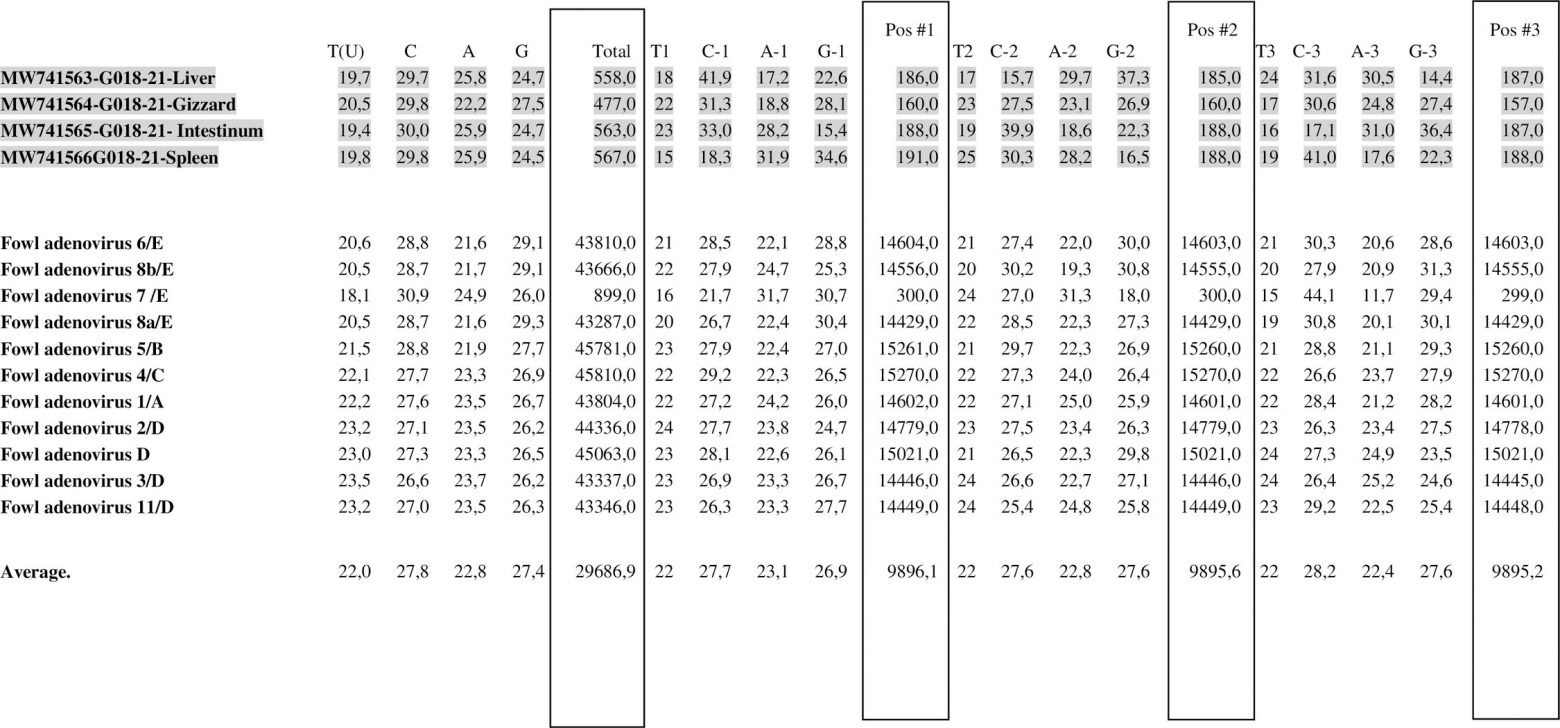
Analysis of codon composition of clinical strain sequences isolated from broiler chickens indicated in grey. Total number of nucleotides of examined strain sequences, number of nucleotides in the sequences tested in the first, second and third codon positions, respectively.

Analysis of the codon composition of the examined strains has been presented in (Fig 2).

### Analysis of the number of successive codons and relative synonymous codon usage

**Fig 3:**
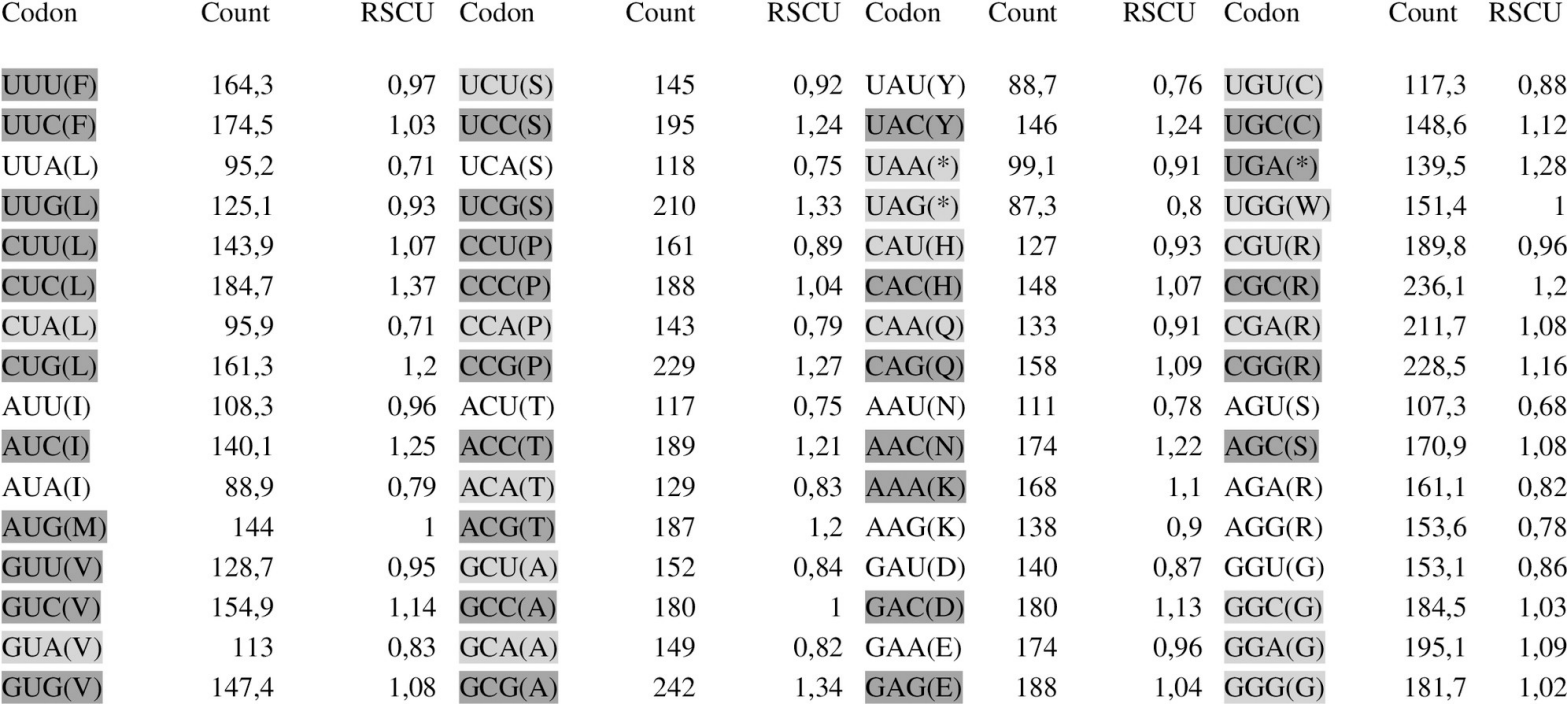
Relative synonymous codon usage (RSCU) for clinical FAdVs strain sequences. Darker shading indicates a higher RSCU value.

All frequencies are given in percentages. The colour intensity indicates how highly an amino acid is preferred in a particular position amongst examined strain sequences. Darker shadings indicate a higher RSCU value. The value of the average codons is 9893 in codon usage bias (Fig 3).

The codon usage in the Loop L1 region of the hexon gene sequences from the field and reference fowl adenovirus strain sequences were examined. C (cytosine) was the most frequent nucleotide for adenovirus field sequences type/species 1/A and 11/D ranging from 29.7% to 30.0% compared with reference strain sequences which ranged from 26.6% to 30.9%. Additionally, C (cytosine) appeared most often in the first position of the codon in all examined sequences, and the percentages were estimated to be between 18.3% to 41.9%. T (thymine) was the least common nucleotide for each examined adenovirus type/species ranging from 17% to 25% compared with reference strain sequences which ranged from 16% to 24%.

### Tajima’s neutrality test

Tajima’s neutrality test has been performed and the results were presented in Table 1. The research studies involved 15 nucleotide fowl adenovirus sequences. Codon positions integrated 1st+2nd+3rd and noncoding. All invalid sequences with gaps and missing data have been removed. In the final dataset of 470 positions were examined at list. *Abbreviations*: *m* = number of sequences, *n* = total number of sites, *S* = Number of segregating sites, *p*_s_ = *S*/*n*, *Θ* = *p*_s_/a_1_, *π* = nucleotide diversity, and *D* is the Tajima test statistic [48].

**Table 1:** Tajima’s neutrality test.

*m*	*S*	*P*_*s*_	*Q*	*π*	*D*
15	470	1. 000000	0.307544	0,672888	5.282195

## Discussion

This study characterizes the coinfection of circulating FAdVs type/species 1/A and 11/D with ARVs in poultry flock in Poland. In poultry, this combination accounts for several clinical instances of disease in Poland. FAdVs and ARVs are important from an epidemiological point of view poultry viruses that can induce disease and are strictly related to immunosuppression in birds. Previous research has shown that some adenovirus species are more virulent and are capable of causing several diseases. Further, some of these diseases have been reported to result in high rates of mortality. Specifically, FAdVs 8a/E and 8b/E, and 11/D were responsible for clinical cases of IBH in several countries such as Canada, Australia, New Zealand, Egypt, and India [14, 17, 22, 25, 33, 49]. The detection and identification of two adenovirus species, A and D, which may influence pathogenesis are still under evaluation.

Worldwide, poultry flocks are affected by vertically transmitted viruses such as chicken infectious anaemia virus (CIAV) and reoviruses (ARVs). These viruses have been shown to induce serious disease that has high economic consequences. In the current study, we have provided clinical data in assessing confirmed IBH and reovirus coinfection in poultry. In this same flock, Fowl adenovirus type/species, 1/A and 11/D were isolated, and additionally, in those chickens, a reovirus infection has been confirmed. Reoviruses were isolated from liver and spleen of affected birds. We investigated possibly other factor which could interact with the ARVs conducting these clinical signs, however no effect.

Hexon and fibre are the main structural proteins of FAdV [50]. The hexon protein is the major capsid protein of the non-enveloped icosahedral virion, where type, group-, and subgroup-specific determinants are located, and Loop L1 region HVR1-4 of the hexon gen have been used for the studies [12, 51].

Research studies conducted by Fadly, 1976 [31] and Rosenberger, 1975 [52] indicated that IBDV-induced immunosuppression and facilitates with IBH coinfection. A similar observation was made in New Zealand, where IBH is the most commonly occurring pathogen in chickens [14, 50, 53]. Concerning infections with CIA and other adenoviruses, Von Bulow, 1986 [54] indicated that reovirus infection was associated with an increased incidence of hepatitis and death of affected chickens. However, in India, IBH was associated with the presence of aflatoxins in the feed [27]. In studies conducted in Trinidad and Tobago, three viruses have been identified (FAdV with three type/species designed as 8a/E, 8b/E, and 11/D) CIAV and IBDV have been participated in the coinfection. These adenoviruses which represent two species, D and E, are the most prevalent in Trinidad and Tobago today [55]. Adenoviruses and reoviruses were also isolated from pigeons and mallard ducks [25]. In early 2019, an epidemiologic survey on poultry flocks in eight commercial broiler farms in China showed that FAdV-4 and ARVs have a high coinfection rate. The data showed that this coinfection accounted for 63% of all ARV-positive samples [28]. Yan, 2020 provided data showed that FAdV-4/C and ARVs coinfection caused severe damage to the SPF chicken’s immune system. Further, these findings provide useful insights into the pathology, prevention, and treatment of FAdV-4/C and ARVs coinfection. Roussan, 2012 [56] examined poultry flocks in Jordan, he found that 4.3% and 2.7% of these flocks were positive for astrovirus, reovirus, and adenovirus infection, respectively. In 1998, Lenz [57] showed that adenovirus strains were more pathogenic than the reovirus strains in the digestive system. More recently, El-Tholoth, 2020 [22] indicated that the coinfection of adenoviruses of species D, mainly with IBD and CIA, is an indicator of immunodeficiency in chickens.

This study aimed to identify the molecular characteristics of adenovirus strains isolated from birds with clinical reovirus infection.

## Conclusions

The efficient and accurate identification and monitoring of coinfections/superinfections in the field are important. Molecular tools such as PCR are more accurate and less sensitive than seroprevalence strategies. Further, seroprevalence approaches are non-specific for adenovirus type/species and their co-infective pathogens. As there is no commercial vaccine against FAdVs in Poland, understanding the direct and indirect interactions of reoviruses and adenoviruses, both during coinfection as well as the individual roles of pathogens, is crucial to investigate in the future.
